# Ovarian toxicity of e-cigarette liquids: Effects of components and high and low nicotine concentration e-cigarette liquid *in vitro*

**DOI:** 10.18332/tid/170631

**Published:** 2023-10-09

**Authors:** Tairen Chen, Mengjing Wu, Yuting Dong, Hehe Ren, Meiling Wang, Bin Kong, Yufang Cai, Changchun Hei, Kai Wu, Chengjun Zhao, Yiwei Li, Yucheng Fan, Qing Chang

**Affiliations:** 1 Key Laboratory of Fertility Preservation and Maintenance of Ministry of Education, School of Basic Medical Sciences, Ningxia Medical University, Yinchuan, China; 2 Department of Critical Care Medicine, General Hospital of Benxi Iron and Steel Industry Group, Liaoning Health Industry Group, Benxi, China; 3 Department of Anatomy, Basic Medical College, Shanxi Medical University, Taiyuan, China; 4 Department of Pathology, The First People's Hospital of Shizuishan, Ningxia Medical University, Shizuishan, China

**Keywords:** ovary, ROS, e-cigarette, toxicology *in vitro*, apoptosis

## Abstract

**INTRODUCTION:**

Electronic cigarette use has become increasingly popular, with potential consequences for reproductive health. We aimed to investigate the effects of different components of e-liquid on the ovary and compare the impact of low nicotine concentration e-liquids (LN e-liquids) and high nicotine concentration e-liquids (HN e-liquids) on ovarian toxicity.

**METHODS:**

A total of 378 rat ovaries were divided into seven groups, including control (no intervention), nicotine (0.05 mg/mL), flavoring (0.25 μL/mL), propylene glycol (PG) (2.5 μL/mL), vegetable glycerin (VG) (2.0 μL/mL), LN e-liquid (0.05 mg nicotine + 0.25 μL flavoring + 2.5 μL PG + 2.0 μL VG + 0.25 μL distilled water/mL medium) and HN e-liquid groups (0.05 mg nicotine + 0.05 μL flavoring + 0.5 μL PG + 0.4 μL VG + 0.05 μL distilled water/mL medium). After three hours of in vitro culture, ovarian morphology, oxidation levels [superoxide dismutase (SOD), catalase (CAT), glutathione peroxidase (GSH-Px) and malondialdehyde (MDA)], and apoptosis levels [factor related apoptosis (Fas), Cyt-c, Caspase-9, Caspase-3] were analyzed.

**RESULTS:**

Our findings indicate that nicotine has limited impact on the ovary, while flavoring, PG, and VG all cause ovarian damage including morphological damage, disruption of oxidative balance and promotion of apoptosis, with VG having the most significant effect. Moreover, LN e-liquids may lead to more severe ovarian damage than HN e-liquids at an equal intake of total nicotine.

**CONCLUSIONS:**

Our study highlights that in e-liquid formula, nicotine has a limited effect on the ovaries, but flavoring, PG, and VG all cause damage to the ovaries, with VG the most damaging. At a consistent level of total nicotine intake, e-liquids with low nicotine concentrations cause more damage to the ovaries than those with high nicotine concentrations. These findings contribute to a better understanding of the impact of e-liquids on ovarian health and have important implications for public health policy.

## INTRODUCTION

Tobacco smoking is a major public health issue worldwide, and it is estimated that 1 billion people will die from tobacco-related diseases in the 21st century if current trends continue^1^. Nicotine, the primary addictive substance in tobacco, is ingested by people primarily through smoking cigarettes. However, smoking tobacco also releases a large number of toxic substances that have been shown to cause damage to various organs, including the ovaries^2,3^. Smoking cessation is already a global consensus, and the use of e-cigarettes as a potential alternative to conventional tobacco for smoking cessation has gained popularity due to its lower harm to the human body and the wide range of flavors and nicotine concentrations that can be used^4^.

Electronic cigarettes (e-cigarettes) are devices that consist of a cigarette stick that utilizes a battery and a nebulizer, which heats up an e-cigarette liquid (e-liquid) usually composed of nicotine, vegetable glycerin (VG), propylene glycol (PG), and various types of flavoring agents. The e-liquid is aerosolized during use, and the nicotine is inhaled into the body along with the aerosol^5^. While much research has been conducted on e-cigarettes, it has been suggested that e-liquids themselves can damage tissues *in vitro*, inducing tissue oxidation and disrupting steroidogenesis^6,7^. Furthermore, PG and VG can induce inflammatory responses in gingival epithelial cells, and flavorings prepared from different chemical components also have toxic effects^8,9^. Previous studies have found that e-liquids have toxic effects on the ovary *in vitro*^10^, but the impact of various components on the ovary *in vivo* has yet to be explored.

With the emergence of e-liquids with low nicotine concentration (LN) in recent years, legislation has been passed to limit the maximum nicotine concentration in e-liquids to 20 mg/mL^11^. It has been reported that smokers using LN e-liquids consume more e-liquids more frequently and in larger quantities to compensate for the decreased nicotine content, while smokers using e-liquids with higher nicotine concentration (HN) require less inhalation^11^. As such, it is worthwhile to explore whether smoking LN e-liquids (relatively more e-liquid intake) has more toxic effects on the ovaries compared to smoking HN e-liquids (relatively less e-liquid intake), in the presence of an equal amount of nicotine intake. Hence, this study aims to investigate the effects of different components of e-liquids on the ovaries *in vitro* to explore whether LN e-liquids cause more damage to the ovaries than HN e-liquids *in vitro*.

## METHODS

### Animals

A total of 189, 35-day-old SD female rats (100 ± 15 g) were obtained from the Laboratory Animal Center of Ningxia Medical University, with a Certificate of Conformity No: SCXK (Ning) 2021-0001. The rats were housed under standard laboratory conditions with a temperature of 22–24°C and a 12-h light/dark cycle with *ad libitum* access to water and standard rodent chow. The animal experiment was conducted in accordance with the Guidelines for Ethical Review of Laboratory Animal Welfare issued by the General Administration of Quality Supervision, Inspection and Quarantine and the National Standardization Administration. The experiment was repeated at least three times.

### Materials

Based on previously published protocols, we formulated two types of e-liquids to meet our experimental needs^6,7,10^. For the 1mL LN e-liquid, we used a solution of nicotine salt [10 mg dissolved in propylene glycol (PG)], vegetable glycerin (VG) (40%, 400 μL), PG (50%, 500 μL), distilled water (5%, 50 μL), and tobacco flavoring agent (5%, 50 μL). For the 1 mL HN e-liquid, we used a solution of nicotine salt (50 mg dissolved in PG), VG (40%, 400 μL), PG (50%, 500 μL), distilled water (5%, 50 μL), and tobacco flavoring agent (5%, 50 μL). We obtained nicotine salt (99.9%) from Hubei Nuohe, China, VG (PRB-297545V) and PG (PRB-362789P) from Probase (China), and tobacco flavoring agent from Prolavor (China).

### Sample preparation

The culture medium was prepared by mixing F12 and DMEM at a 1:1 ratio, supplemented with 12% fetal bovine serum (Gibco). The 35-day-old female Sprague-Dawley rats were identified as being in puberty and their estrous cycles were determined by vaginal smear. Rats in the diestrus phase were selected for the experiment^12^. The rats were euthanized by cervical vertebra dislocation and the dorsal region of the kidney was sterilized with 75% alcohol. The ovaries were then removed and divided into seven groups for intervention culture: control (no intervention), nicotine, flavoring, PG, VG, LN e-liquid, and HN e-liquid groups.

The intervention plan was as follows: the concentrations of flavoring, PG and VG were determined according to the concentration of nicotine. Following previous experiments using a dose of 0.5 mg/kg of nicotine^6,7,13^, a nicotine concentration of 0.05 mg/mL of medium was used to interfere with the ovaries in the nicotine group. For the VG, PG, and flavoring groups, 2 μL of VG/mL, 2.5 μL of PG/mL, and 0.25 μL of flavoring/mL were added to the medium, respectively. The intervention components of the LN e-liquid group were as follows: 0.05 mg/mL of nicotine, 0.25 μL of flavoring/mL, 2.5 μL of PG/mL, 2 μL of VG/mL, and 0.25 μL of distilled water/mL of medium. The intervention components of the HN e-liquid group were as follows: 0.05 mg/mL of nicotine, 0.05 μL of flavoring/mL, 0.5 μL of PG/mL, 0.4 μL of VG/mL, and 0.05 μL of distilled water/mL of medium.

The ovaries were then incubated in a CO_2_ incubator at 37°C for 3 h, based on previous studies^10^, and were subsequently rinsed with PBS to terminate the intervention. After the intervention, some ovaries were fixed with 4% paraformaldehyde solution for 12 h for morphological observation, while the other ovaries were stored at -80^o^C until the biochemical analysis and ELISA studies were conducted.

### Morphological observations

HE staining of the ovaries of each experimental group was conducted to observe the morphological impact of intervention on the ovaries of each experimental group. PBS solution was used for washing at the end of the incubation and proceeded to the routine fixation, dehydration, embedding, and sectioning procedure. Ovary sections were controlled at a thickness of 5 μm, and one was selected every 6 sections, and 10 sections were collected from each ovary for staining observation (eosin for 3 min and hematoxylin for 1 min, and a 5 s differentiation was produced in HCl alcohol after hematoxylin staining). At the end of staining, routine sealing was performed, and the sections were observed under a light microscope, and suitable fields were selected for taking photographs. The judgment criteria of healthy and atretic follicles were referred to previous reports^14,15^. The experiments were repeated three times using 18 ovaries per group, for a total of 126 ovaries.

### Oxidative stress assessment

The ovaries were weighed and then homogenized as tissue homogenates. A volume of 9 mL of physiological saline was added, using a ratio of weight (g) to volume (mL) of 1:9 (1 g of ovarian tissue to 9 mL of physiological saline), followed by tissue homogenization. The tissue homogenates were then centrifuged at 3000 rpm/min for 15 min, after which, the supernatant was aspirated to detect superoxide dismutase (SOD), catalase (CAT), glutathione peroxidase (GSH-Px), and malondialdehyde (MDA), according to the instructions on the product. The experiments were repeated three times using 18 ovaries per group, for a total of 126 ovaries.

*Determination of SOD*

SOD plays a crucial role in maintaining the oxidative and antioxidant balance of the organism. This enzyme scavenges superoxide anion free liquid (O_2_ - •) to protect cells from damage. By measuring the activity of SOD, one can determine the body’s antioxidant capacity and oxidative balance^16^. The total SOD assay kit (Nanjing Jiancheng, a001-1) was used in this experiment to measure SOD activity (U/mg prot) in ovarian homogenates of each group, following the instructions.

*Determination of CAT*

SOD rapidly dismutates superoxide anion free liquid (O_2_ - •) to less dangerous H_2_O_2_, and CAT further degrades H_2_O_2_ to water and oxygen. Therefore, measuring the activity of CAT in tissues can also reflect the body’s antioxidant capacity and oxidative balance^17^. The CAT assay kit (Nanjing Jiancheng, a007-1) was used to detect CAT activity (U/mg prot) in the ovarian homogenates of each group, following the instructions.

*Determination of GSH-Px*

GSH-Px is an important enzyme that catalyzes the decomposition of H_2_O_2_ and is widely present in the body. Its activity can play a role in protecting the structural and functional integrity of the cell membrane. Therefore, measuring the activity of GSH-Px in tissues may reflect the antioxidant capacity and oxidative balance of the body^18^. In this experiment, the activity of GSH-Px in ovarian homogenates of each group was determined using a GSH-Px test kit (Nanjing Jiancheng, a005-1), following the instructions.

*Determination of MDA*

The body produces oxygen-free fluid through the enzyme system, which can attack polyunsaturated fats in biological membranes, trigger lipid peroxidation, cause cell damage, and form lipid peroxides, such as MDA. Therefore, the concentration of MDA tested in the body may reflect the degree of lipid peroxidation and indirectly respond to the degree of cell damage^18^. In this experiment, the concentration of MDA in ovarian tissue of each group was detected using the TBA method MAD detection kit (Nanjing Jiancheng, a003-1). After preparing ovarian tissues into tissue homogenates, the experiments were performed following the kit instructions, and the concentration of MDA per mg protein (nmol/mg prot) was calculated.

### Detection of ovarian apoptosis

The ovaries were weighed and prepared as tissue homogenates. The ovarian tissue was added to 20 volumes of lysis buffer at a ratio of weight (g) to volume (mL) of 1:20 (1 g ovarian tissue added to 20 mL lysis buffer). Tissue homogenization was performed, followed by centrifugation at 3000 rpm/min for 15 min. The supernatant was collected to detect apoptotic factors related to Fas (Factor Related Apoptosis), cytochrome-c (Cyt-c), caspase-9 (CASP9), and caspase-3(CASP3) following the instructions provided by the respective assay kits. Experiments were performed in triplicate using 18 ovaries per group, with a total of 126 ovaries analyzed.

*Detection of Fas*

Fas is a death receptor protein that triggers apoptosis in various tissues, including the ovary. Elevated Fas expression is associated with increased cell death in the ovary. We measured Fas levels in ovarian homogenates of each group using an enzyme-linked immunosorbent assay (ELISA) kit (sea030ra, CLOUD-CLONE CORP, Wuhan, China) following the manufacturer’s instructions.

*Detection of Cyt-c, CASP9, CASP3*

Cyt-c, CASP9, and CASP3 are major proteases involved in the mitochondria-mediated cell death pathway. Apoptotic stimuli trigger the release of these factors into the cytosol, where they activate a cascade of events that ultimately lead to cell death. We measured levels of Cyt-c, CASP9, and CASP3 in ovarian homogenates of each group using ELISA kits (sea594ra for Cyt-c, sea627ra for CASP9, and sea626ra for CASP3, all from CLOUD-CLONE CORP, Wuhan, China) according to the manufacturer’s instructions.

### Statistical methods

All experimental data are shown as mean ± SD using analysis of variance (ANOVA) followed by Fisher’s least significant difference using SPSS software for test (Fisher LSD). Statistical differences were considered significant when p<0.05.

## RESULTS

### Ovarian follicle morphology assessment

In the control group, the ovarian follicle morphology was normal, with a regular oval shape, and the granulosa cells (GCs) were arranged tightly and neatly. There was less nuclear pyknosis, and both the GC layer and the theca cell layer were intact, healthy, and closely connected with the GCs of follicles.

**Figure 1:**
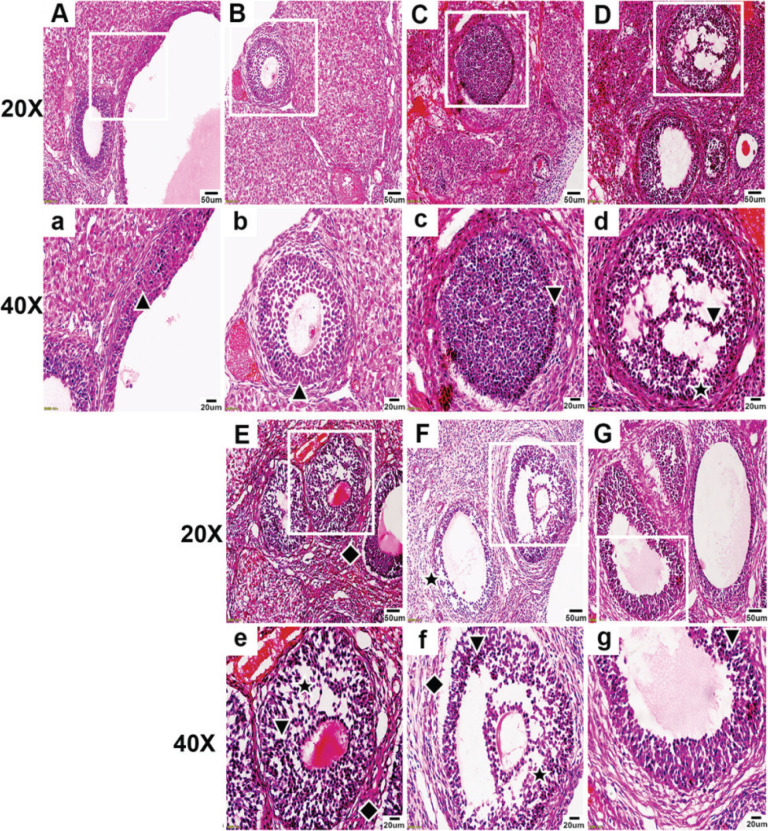
Under 20× and 40× microscopy, HE staining of ovaries was observed in the control, nicotine, flavoring, PG, VG, LN e-liquid, and HN e-liquid groups. The normal granulosa cell (GC) layer is indicated by ▲, the abnormal GC layer by ▼, GC loss by ★, and separation of the GC layer from the theca cell layer by ◆

In the nicotine group, the follicle morphology and GC arrangement were normal, with less nuclear pyknosis, and the theca cell layer was intact and healthy and closely connected with GCs (Figure 1).

In the flavoring group, the follicle morphology appeared abnormal, and the GC layer and the theca cell layer near the theca cell were both abnormal, with signs of detachment from the GC layer.

In the PG group, the follicle morphology appeared abnormal, with a large area of abnormalities in the GC layer, accompanied by the phenomenon of GC loss, and a small part of the theca cell layer appeared separated from the GC layer (Figure 1).

In the VG group, the follicle morphology appeared markedly abnormal, with an irregular oval shape, and the GC layer showed a large area of abnormality, arranged in a scattered and irregular pattern, accompanied by a large area of GC loss. The theca appeared obviously abnormal, scattered, irregular, and the cells were lost, and most of the theca had separation from the GC layer (Figure 1).

In the LN e-liquid group, the follicle morphology appeared abnormal, with a large area of abnormal GC layer arranged in a disorganized and irregular pattern, accompanied by a large area of GC loss. The theca appeared significantly abnormal, scattered, irregular, and most of the theca cells were lost, and the theca appeared clearly separated from the GC layer.

In the HN e-liquid group, the follicle morphology appeared abnormal, with some GC layers appearing abnormal, but the arrangement of most GCs was more neat and compact than the LN e-liquid group, and the loss of GCs was less obvious than the LN e-liquid group. The theca appeared abnormal with loss of theca cells and was separated from the GC layer, but much less separation than the LN e-liquid group (Figure 1).

### Oxidative stress assessment

**Figure 2:**
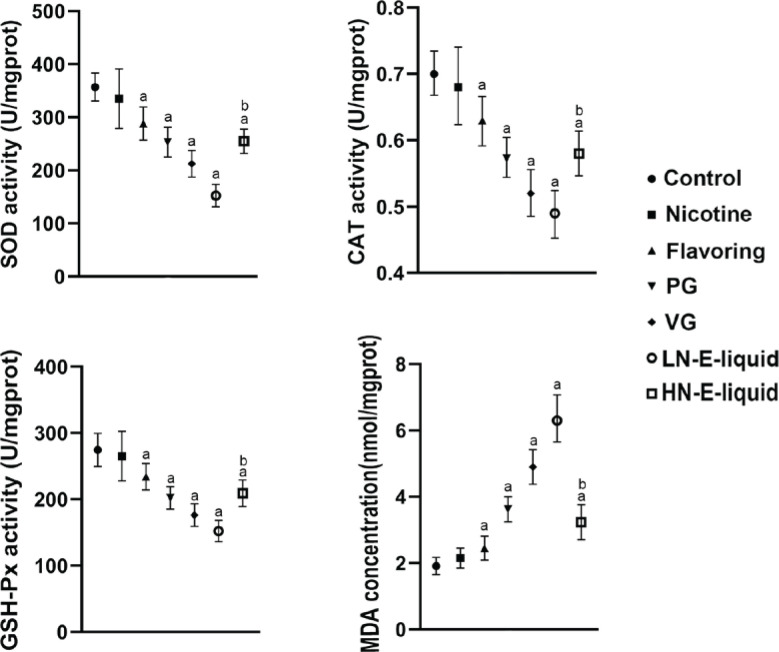
SOD, CAT, GSH-Px activity and MDA content of each group: a) compared with the control group, p<0.05; b) compared with the LN e-liquid group, p<0.05

The results showed that compared with the control group, there were no significant differences in SOD, CAT, and GSH-Px in the nicotine group. However, in the flavoring, PG, VG, and LN e-liquid groups, there was a decrease in these antioxidant enzymes, with MDA elevated in these groups. In the HN e-liquid group, there was a decrease in SOD, CAT, and GSH-Px, and MDA was elevated, but was significantly lower compared to the LN e-liquid group (Figure 2).

### Apoptosis assessment of each group

**Figure 3:**
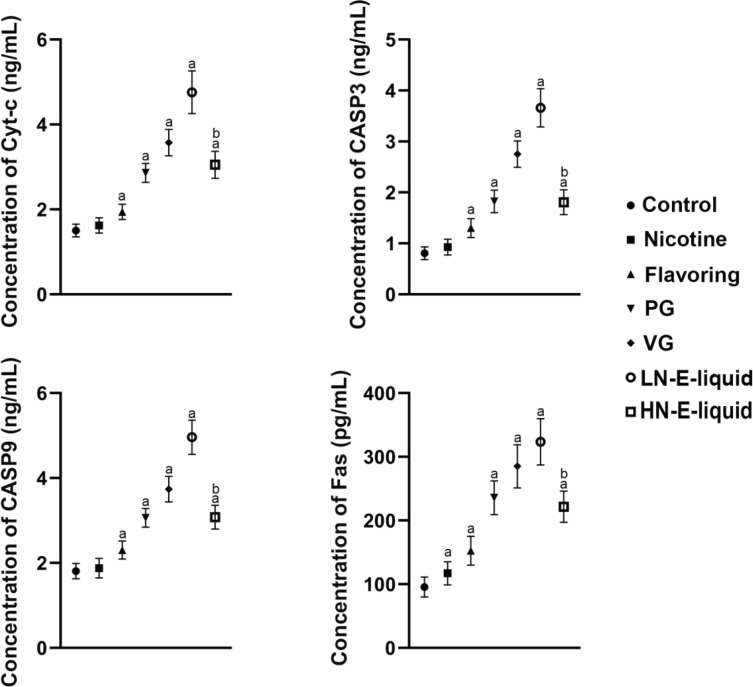
Cyt-c, CASP3, CASP9, Fas contents in each group: a) compared with control group p<0.05; b) compared with LN e-liquid p<0.05

The results showed that compared with the control group, there were no significant differences in Cyc-c, CASP3, and CASP9 in the nicotine group, but Fas was elevated. Cyc-c, CASP3, CASP9, and Fas were all increased in a stepwise manner in the flavoring, PG, VG, and LN e-liquid groups. In the HN e-liquid group, they were all elevated, but were significantly lower compared with the LN e-liquid group (Figure 3).

## DISCUSSION

The present study investigated the effects of individual components in e-liquid on ovary morphology, oxidative balance, and apoptosis. Overall, the findings indicate that the ingestion of e-liquids containing various components can significantly impact the ovary, both in terms of morphology and function.

In terms of ovary morphology, our results suggest that nicotine, on its own, has limited effects. However, in the presence of flavoring, PG, and VG, we observed abnormal follicle morphology, with the greatest damage found in the VG group, followed by PG. LN e-liquid was found to have a harsh impact on follicle morphology, whereas the effect of HN e-liquid was less severe. This may be due to differences in the intake of flavoring, PG, and VG between the two groups.

Our biochemical analysis of ovary tissue also revealed significant effects on oxidative balance^16-18^. Specifically, we observed an increase in active oxygen levels and a reduction in antioxidant capacity in the presence of flavoring, PG, and VG. VG had the most significant impact on oxidative balance, followed by PG. Similarly, LN e-liquid was found to exacerbate the ovarian oxidation level compared to HN e-liquid.

Increased oxidation can, in turn, lead to apoptosis, and our study found that cell death occurred in the ovaries of each group due to the increased Fas, Cyc-c, CASP3, and CASP9 expression^19,20^. Of note, VG had the most significant impact on apoptosis, followed by PG. In contrast, nicotine had a limited impact on ovarian apoptosis, although we did observe increased Fas expression in this group. Both LN e-liquid and HN e-liquid were found to induce apoptosis, with LN e-liquid causing more severe damage.

### Limitations

We must take into account the limitations of our study. This study conducted an experiment *in vitro* to observe the toxicity of each component of the e-liquid to the ovary, and judge the toxicity of e-liquid with high and low nicotine content to the ovary when the nicotine concentration is consistent. However, it needs to be noted that cellular exposure differs from human exposure due to inhalation, and therefore the results should be interpreted with caution. In the following research, we plan to further carry out the inhalation experiment *in vivo*.

## CONCLUSIONS

Our study highlights the potential risks associated with the use of e-liquid. While nicotine has limited influence on the ovary, our findings suggest that flavoring, PG and VG in e-liquid can all cause damage to the ovary, with VG causing the most severe damage. Furthermore, we found that LN e-liquid may cause more damage to the ovary than HN e-liquid when the total amount of nicotine intake is consistent. This underscores the fact that the level of nicotine in e-liquid is not the only important factor to consider when evaluating its safety. Overall, our study provides important insights into the potential dangers of e-liquid use and has important implications for public health. Further research is needed to better understand the long-term effects of e-liquid on the human body and to identify ways to mitigate its potential harms.

## Data Availability

The data supporting this research are available from the authors on reasonable request.
